# Histamine 1 Receptor Blockade Enhances Eosinophil-Mediated Clearance of Adult Filarial Worms

**DOI:** 10.1371/journal.pntd.0003932

**Published:** 2015-07-23

**Authors:** Ellen Mueller Fox, Christopher P. Morris, Marc P. Hübner, Edward Mitre

**Affiliations:** 1 Department of Microbiology and Immunology, Uniformed Services University, Bethesda, Maryland, United States of America; 2 Institute for Medical Microbiology, Immunology, and Parasitology, University Hospital Bonn, Bonn, Germany; University of Liverpool, UNITED KINGDOM

## Abstract

Filariae are tissue-invasive nematodes that cause diseases such as elephantiasis and river blindness. The goal of this study was to characterize the role of histamine during *Litomosoides sigmodontis* infection of BALB/c mice, a murine model of filariasis. Time course studies demonstrated that while expression of histidine decarboxylase mRNA increases throughout 12 weeks of infection, serum levels of histamine exhibit two peaks—one 30 minutes after primary infection and one 8 weeks later. Interestingly, mice treated with fexofenadine, a histamine receptor 1 inhibitor, demonstrated significantly reduced worm burden in infected mice compared to untreated infected controls. Although fexofenadine-treated mice had decreased antigen-specific IgE levels as well as lower splenocyte IL-5 and IFNγ production, they exhibited a greater than fourfold rise in eosinophil numbers at the tissue site where adult *L*. *sigmodontis* worms reside. Fexofenadine-mediated clearance of *L*. *sigmodontis* worms was dependent on host eosinophils, as fexofenadine did not decrease worm burdens in eosinophil-deficient dblGATA mice. These findings suggest that histamine release induced by tissue invasive helminths may aid parasite survival by diminishing eosinophilic responses. Further, these results raise the possibility that combining H1 receptor inhibitors with current anthelmintics may improve treatment efficacy for filariae and other tissue-invasive helminths.

## Author Summary

Filariae are tissue-invasive parasitic roundworms that infect over 100 million people worldwide and cause debilitating conditions such as river blindness and elephantiasis. One of the major factors limiting our ability to eliminate these infections is the lack of drugs that kill adult worms when given as a short course therapy. Additionally, the mechanisms by which adult worms are cleared from infected individuals remains poorly understood. In this study, we demonstrate that treatment of infected mice with fexofenadine, an inhibitor of histamine receptor 1, significantly reduces adult worm numbers through a mechanism dependent on host eosinophils. These findings suggest that histamine release induced by parasitic worms may aid parasite survival by decreasing eosinophilic responses. Further, as antihistamines are generally safe medications, these results raise the possibility that antihistamine therapy may be useful either alone, or potentially in combination with other antifilarial medications such as diethylcarbamazine (DEC), to eliminate adult filarial worms from infected individuals.

## Introduction

Filariae are vector-borne tissue-invasive nematodes that infect over 100 million people worldwide and cause the debilitating conditions of river blindness and elephantiasis [1]. A major obstacle to ongoing efforts to control and potentially eradicate these diseases is the limited ability of anti-filarial drugs to kill adult worms, especially when given as single dose treatments.

One of the fairly unique aspects of helminth infections, in contrast to infection with most other pathogens, is the induction of histamine release in response to the parasites. Like other helminths, filariae induce the production of antigen-specific IgE, which then sensitizes basophils and mast cells to release histamine in response to parasite antigens. Histamine (2-[4-imidazolyl]ethylamine) is a short-acting biogenic amine that, in addition to having potent acute inflammatory properties, also has numerous immunomodulatory effects on chronic inflammation [2]. Histamine is synthesized by the enzyme histamine decarboxylase (HDC) and is either stored in cytoplasmic granules in basophils and mast cells or is immediately released into the periphery [3]. Histamine release from both basophils and mast cells in response to parasite antigen has been observed in numerous studies of helminth infections [2,4–8]. Although sensitivity to parasite antigens is primarily dependent on parasite-specific IgE [9], several helminths can also induce histamine release in the absence of parasite-specific IgE [10]. In this study we investigated the role histamine plays in the immune response to filariae and the effect antihistamine therapy has on filarial worm burdens. Using the *Litomosoides sigmodontis*/mouse model we observed that administration of fexofenadine, a histamine receptor 1 antagonist (HR1i), reduces adult worm numbers by over 50%. Additionally, clearance of adult worms in HR1i treated mice was found to be primarily eosinophil dependent, as HR1i administration did not enhance worm clearance in eosinophil-deficient infected mice.

## Materials and Methods

### Ethics statement

All experiments were performed under protocols approved by the Uniformed Services University Institutional Animal Care and Use Committee.

### Mice and parasites

Female BALB/c (NCI, Frederick, MD), and BALB/c eosinophil deficient (ΔdblGATA mice, The Jackson Laboratory, Bar Harbor ME), were maintained at the Uniformed Services University with free access to food and water. At study endpoints, all animals were euthanized using carbon dioxide followed by cervical dislocation. Blood was collected by cardiac puncture.

For *Litomosoides sigmodontis* infection, L3-stage larvae (L3s) were obtained from infected jirds *(Meriones unguiculatus*, TRS labs, Atlanta, GA) by pleural lavage with RPMI 1640. 40 L3s were collected and injected subcutaneously (dorsal neck) into 6–10 week old mice as previously described [11].

For microfilarial counts, 30 μl of blood was taken and mixed with 1 mL ACK lysing buffer (Quality Biological). To count microfilarial numbers, the lysate was pelleted and assessed for counts microscopically.

### Antihistamine administration

Fexofenadine HCl, an HR1 antagonist (HR1i), was dissolved in the drinking water at a concentration of 0.25 mg/ml for an average daily dosage of 20mg/kg/day. Cimetidine (Sigma-Aldrich), an HR2 antagonist, was prepared by dissolving in hydrochloric acid (HCl) and mixed with water. The pH was then adjusted to 7.0 with sodium hydroxide (NaOH). The final concentration of cimetidine was 2.5 mg/ml in drinking water, for an average daily dosage of 200 mg/kg/day. Drinking water bottles containing antihistamines were changed every other day. Antihistamine activity was confirmed by testing stomach pH at time of euthanasia (for HR1 antagonists) and by local anaphylaxis in response to a direct histamine challenge (for HR2 antagonists).

### Histamine ELISA

Blood was collected at different time points in heparinized plasma separator microfuge tubes (Starstedt, Nümbrecht, Germany). Samples were centrifuged at 15,000 X g for 1.5 minutes. Histamine in plasma was detected using a commercially available histamine ELISA assay according to the manufacturer’s instructions (Beckman-Coulter).

### Preparation of histological sections

Adult worms were collected from the pleural cavity of infected animals at 8 weeks post infection. Adult worms were fixed overnight in 4% paraformaldehyde and were washed in 70% ethanol prior to histological processing by Histoserv, Inc (Rockville, MD). In brief, the fixed tissue was dehydrated through graded alcohols, cleared in xylene and infiltrated with paraffin. The processed tissue was then embedded in paraffin and sectioned on a microtome at 5 microns. The slides were then deparaffinized in xylene, hydrated through graded alcohols to water then stained with Carazzi’s hematoxylin. Following a water rinse, they were stained with eosin and dehydrated with graded alcohols. The slides were then cleared using xylene and coverslipped with permount.

### RT-PCR to detect HDC synthesis

RNA from whole blood was isolated according to the manufacturer’s instructions (Ambion, Mouse Whole Blood RNA isolation). cDNA synthesis was performed using random primers according to the manufacturer’s instructions (iScript cDNA synthesis kit, BioRad). RT-PCR was performed using a murine histidine decarboxylase (HDC) gene expression assay following manufacturer’s instructions. Samples were analyzed using an Applied Biosystems 7500 Real-Time PCR system and results calculated as fold change relative to an endogenous 18s rRNA control using the 2^-ΔΔ CT^ method.

### Parasite antigens

*L*. *sigmodontis* worm antigen (LsAg) was prepared as previously described [12].

#### LsAg-specific IgE and total IgE ELISA

All ELISAs were performed using Costar half-area, high-binding plates. For worm-specific IgE, plates were coated with 20 μg/mL *Ls*Ag in PBS and incubated overnight at 4°C. Plates were blocked with PBS/5% BSA and 0.05% Tween 20. IgG was depleted out of plasma using GammaBind plus Sepharose beads (GE Healthcare Biosciences). Plasma was then diluted in 1% BSA/PBS and 5-fold serial dilutions were made. Samples were added to the plate in duplicate. Plates were then washed and plate-bound IgE detected by the addition of biotinylated anti-mouse IgE (BD Biosciences) diluted in 1%BSA/PBS. Following another wash, alkaline phosphatase conjugated streptavidin (Jackson Immuno Research Labs) diluted 1:1000 in 1%BSA/PBS was added. Plates were developed by the addition of 4-nitrophenyl phosphate disodium (4-NPP, Sigma-Alderich) in 0.1M carbonate buffer. Absorbance was read at 405nm using a Victor^3^ V microplate reader from PerkinElmer. Detection of total IgE was performed with identical steps as for parasite-specific IgE except that plates were initially coated with 10 μg/mL anti-IgE (BD Biosciences)in PBS and plasma samples were plated at dilutions of 1:10 and 1:90. Total IgE concentrations were determined using an IgE standard curve (BD Biosciences) and WorkOut 2.0 ELISA software (PerkinElmer).

#### Splenocyte culture and cytokine ELISAs

A single-cell suspension was prepared from spleens by mechanical homogenization and straining through a 22μm cell-strainer. RBCs were lysed using ACK lysis buffer (Quality Biological) and 2.0x10^6^ were plated in IMDM media (Cellgro) containing 1% L-glutamine, 10% FCS, 1% insulin-transferrin-selenium and 80μg/mL gentamicin (Quality Biological). Cells were stimulated with either media, 5μg/mL anti-CD3 and 2μg/mL anti-CD28, or 20μg/mL LsAg and cultured at 37°C, 5% CO_2_ for 3 days. Studies in which purified CD4^+^ and CD11C^+^ were used, cells were magnetically isolated by positive selection according to manufacturer’s instructions (Miltenyi Biotech). Purified CD4^+^ cells were cultured with purified CD11c^+^ cells in a 1:10 ratio (CD11c^+^: CD4^+^). Cells were stimulated as outlined above. ELISAs were performed on culture supernatants. IL-4, IL-5, IFN-γ and IL-10 were detected in culture supernatants using Costar half-area plates and BD OptEIA ELISA kits (BD Biosciences) following the manufacturer’s instructions.

### L3 in vitro survival assay

L3 stage larvae were collected from infected jirds as previously described. For *in vitro* survival assays, 200 L3s were cultured in 5ml of RPMI 1640 medium supplemented with gentamicin. Cultures were supplemented with 200mM histamine or 1mM Fexofenadine HCl and observed daily for mobility to assess survival.

### Flow cytometric identification of eosinophils

To assess eosinophil numbers at site of adult worm infection, pleural cells were collected by pleural lavage. Red blood cells were lysed using ImmunoLyse kit (Beckman Coulter) and then 2.0x10^6^cells/mL were permeabilized with BD Permeablization/Wash buffer (BD Biosciences). For analysis, cells were blocked using CD16/CD32 (soluble FcεR III/II receptor, BD Pharmingen) and stained for flow cytometry using anti-SiglecF PE, anti-CD11c APC and anti-CD45 FITC (all from BD Pharmingen).

Flow cytometry was performed using a BD LSR II system and analyzed with FACSDiVa 6.1 software (BD Biosciences). Antibodies for all flow cytometry experiments were titrated prior to use. During analysis, cut-offs for CD45 positivity and Siglec F positivity were determined using the fluorescence minus one approach.

### Eosinophil-peroxidase (EPO) ELISA

To assess levels of eosinophil peroxidase at the site of adult worm infection, pleural fluid was collected by pleural lavage using 1 mL of sterile RMPI. EPO in lavage fluid was assessed by ELISA according to manufacturers instructions (US Biological Life Sciences).

### Statistical analysis

Statistical analysis was performed using GraphPad Prism software (GraphPad Software, San Diego, Ca). To determine differences between multiple groups, analysis was performed using Kruskal-Wallis test followed by Dunn multiple comparisons. To determine differences between two un-paired groups, Mann-Whitney analysis was performed. A *p* value of <0.05 was considered significant.

## Results

### Infection with Litomosoides sigmodontis results in histamine release in plasma

**Fig 1:**
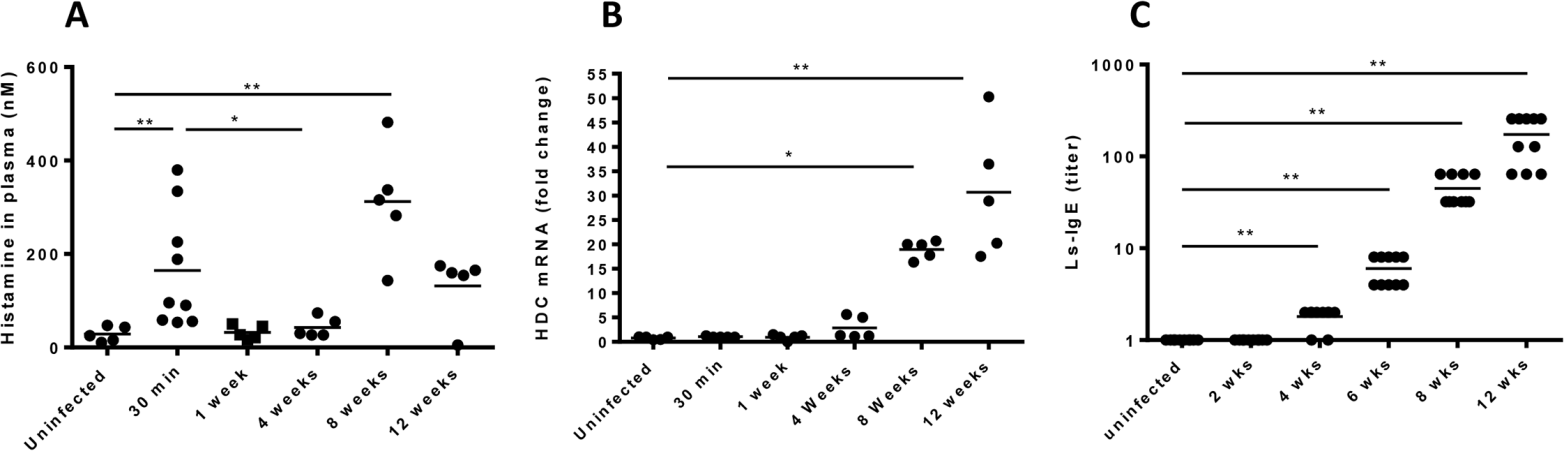
Infection of BALB/c mice with *L*. *sigmondontis* results in two peaks of histamine release. Age-matched BALB/c mice were infected with *L*. *sigmondontis* and assessed for *(A)* Circulating histamine in plasma as measured by histamine ELISA, *(B)* Histidine Decarboxylase (HDC) mRNA from whole blood measured using RT-PCR, fold change relative to uninfected control and *(C)* Parasite-specific IgE titers as measured by ELISA. Data are combined from two independent experiments of 2–3 animals. *p<0.05, **p<0.01, ***p<0.001, Kruskal-Wallis test followed by Dunn Multiple comparison tests.

To determine if infection with *L*. *sigmodontis* results in detectable histamine release, BALB/c mice were infected with 40 L3 stage larvae by subcutaneous injection and circulating histamine levels assessed at 30 minutes, 1, 4, 8 and 12 weeks by competitive ELISA. In this model L3 larvae migrate to the pleural space where they mature to adult worms, start releasing blood-dwelling microfilariae by 7 weeks, and survive for 12–20 weeks. There was a significant peak of circulating histamine observed 30 minutes after injection of L3s and a second, higher peak correlating with the production of microfilariae at 8 weeks of infection (Fig 1A, p < 0.01 for both timepoints when compared to age-matched uninfected controls). Of note, histamine was not detected in the blood of mice 30 minutes after injection of vehicle (sterile RPMI) or peritoneal lavage fluid (S1 Fig).

Using RT-PCR we determined the levels of histidine decarboxylase (HDC) from whole blood RNA. In contrast to histamine, blood levels of HDC mRNA increased throughout the 12 weeks, indicating that histamine may be continually synthesized during infection (Fig 1B). Using ELISA, we determined circulating levels of Ls-specific IgE. Ls-specific IgE levels became detectable after 4 weeks of infection (Fig 1C). Development of detectable Ls-specific IgE thus preceded peak histamine levels in the plasma, suggesting peak histamine release may be due to IgE-mediated basophil and mast cell activation. In contrast, the immediate early release of histamine at the 30 minute time point is suggestive of parasite-specific antibody independent activation of basophils and mast cells.

### Blockade of HR1 and not HR2 results in reduced worm burden

**Fig 2:**
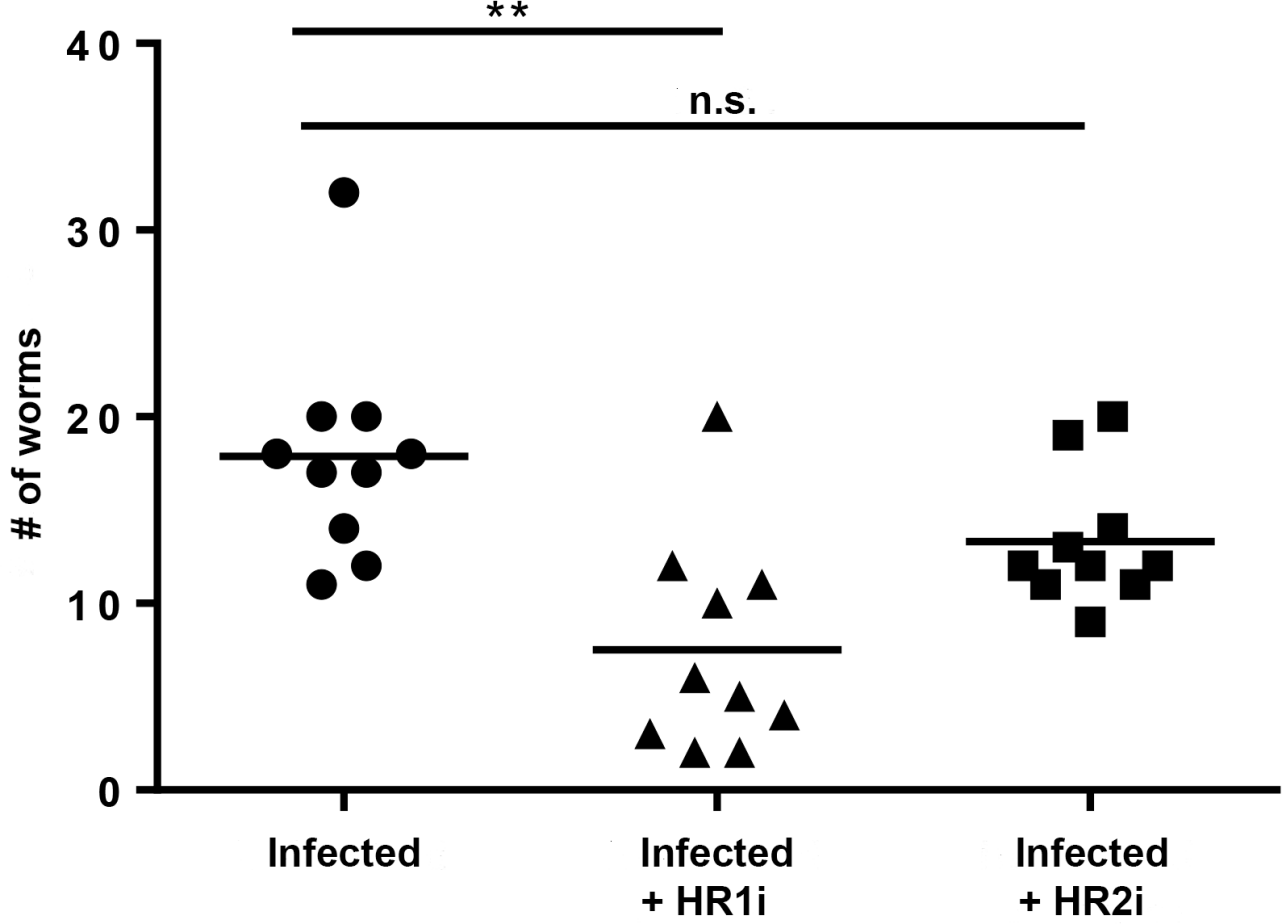
Blockade of HR1 but not HR2 results in reduced adult worm burden. Adult worm burden in BALB/c mice infected with 40 L3s and treated with Fexofenadine (HR1i) or cimetidine (HR2i) for 8 weeks. Data are combined from two independent experiments. **p<0.01, Kruskal-Wallis test followed by Dunn Multiple comparison tests.

We next sought to determine whether histamine plays a role in maintaining worm burdens during primary infection. To test this, BALB/c mice were infected with 40 L3s and treated with HR1 or HR2 antagonists administered in water for the duration of infection. At 8 weeks, mice were euthanized and adult worm burden was determined. Untreated infected mice had a mean recovery of 18 adult worms. Mice treated with HR1 antagonists had a mean recovery of 8 adult worms (58.1% reduction, p = 0.001) while mice treated with HR2 antagonists had a mean recovery of 13 worms (22.5% reduction, p = 0.0573) (Fig 2). This data indicates that signaling via HR1 may play a role in long-term survival of *L*. *sigmondontis* in the mammalian host.

### Blockade of HR1 does not alter microfilariae loads or male-to-female ratio

Given that we observed a reduction of adult worms at 8 weeks post-infection in animals treated with an HR1 antagonist, we sought to determine if HR1 antagonism altered the circulating microfilaria load or the male-to-female ratio. HR1i administration did not alter the number of microfilariae circulating in the blood (S2 Fig) or the male-to-female ratio of recovered adult worms (S3 Fig).The lack of a decrease in microfilaria burden is not too surprising as microfilaria load does not correlate with adult worm numbers [13].

### HR1 blockade enhances adult worm clearance throughout infection

**Fig 3:**
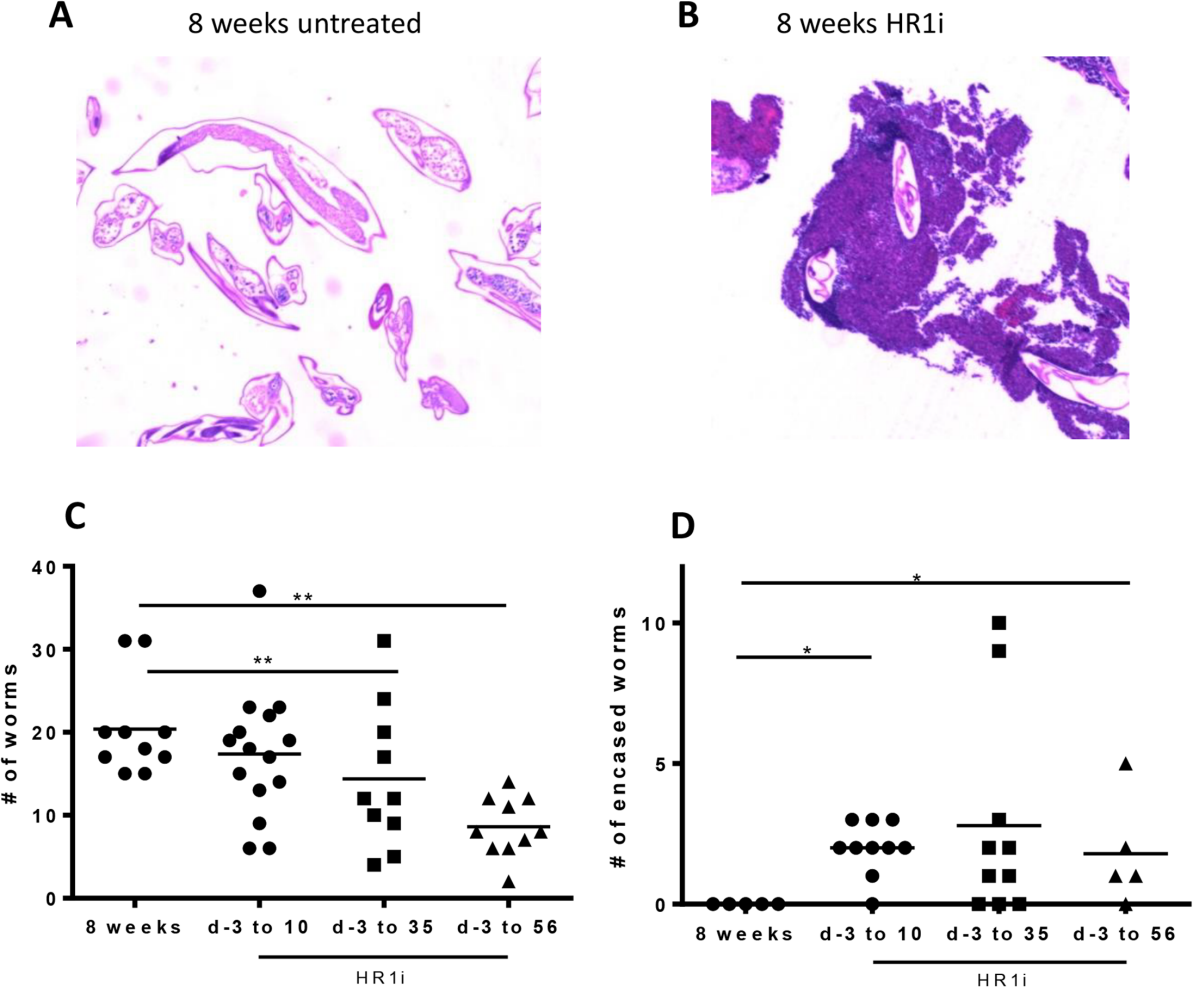
Timing of worm clearance in HR1-treated mice. BALB/c mice were infected with L. sigmodontis by subcutaneous injection of 40 L3 larvae. Fexofenadine (HR1i) was administered in drinking water beginning 3 d prior to infection and continuing until 10, 35, or 56 d after infection. Control mice received no fexofenadine. All mice were euthanized 56 d after infection for enumeration of adult worm burdens. Representative histological sections of A) non-encased and B) granuloma-encased worms stained with hematoxylin and eosin (40x). C) Total numbers of living adult worms (both non-encased and encased) 8 weeks p.i. D) Numbers of encased living worms 8 weeks p.i. Data are combined from two to four independent experiments. *p<0.05, **p<0.01, Kruskal-Wallis test followed by Dunn Multiple comparisons.

Due to the observed reduction in adult worm burdens at 8 weeks, we next sought to determine whether there was a particular timepoint during infection when HR1 blockade enhances worm clearance. In a typical course of infection, L3 stage larvae migrate from the subcutaneous tissues to the pleural space from days 1–5, molt to L4 stage worms by d 11, and then molt to adult worms between days 24–30. Mice were treated for 10, 35, and 56 days post-infection with HR1 antagonist. At 56 days (8 weeks), all groups of mice were euthanized and living adult worms collected. Recovered worms that were motile yet had parts that were covered with granulomas were classified as “encased” (Fig 3A and 3B). As previously demonstrated, mice treated with HR1 antagonists for 8 weeks demonstrated a significant reduction in adult worm burden compared to untreated mice (Fig 3C). While there was no difference in total worm burden at the 8 week timepoint between mice treated with HR1 antagonists for 10 days and untreated mice (mean worm burden of 17 vs 20), mice treated with 10 days of fexofenadine (HR1i) had significantly greater numbers of adult worms that were encased in granulomas (Fig 3D) at 56 days post infection. The trend towards lower worm burdens with longer fexofenadine treatment courses suggests that H1R blockade enhances worm clearance at numerous stages of worm development.

### Histamine and fexofenadine do not have a direct effect on worm survival in vitro

**Fig 4:**
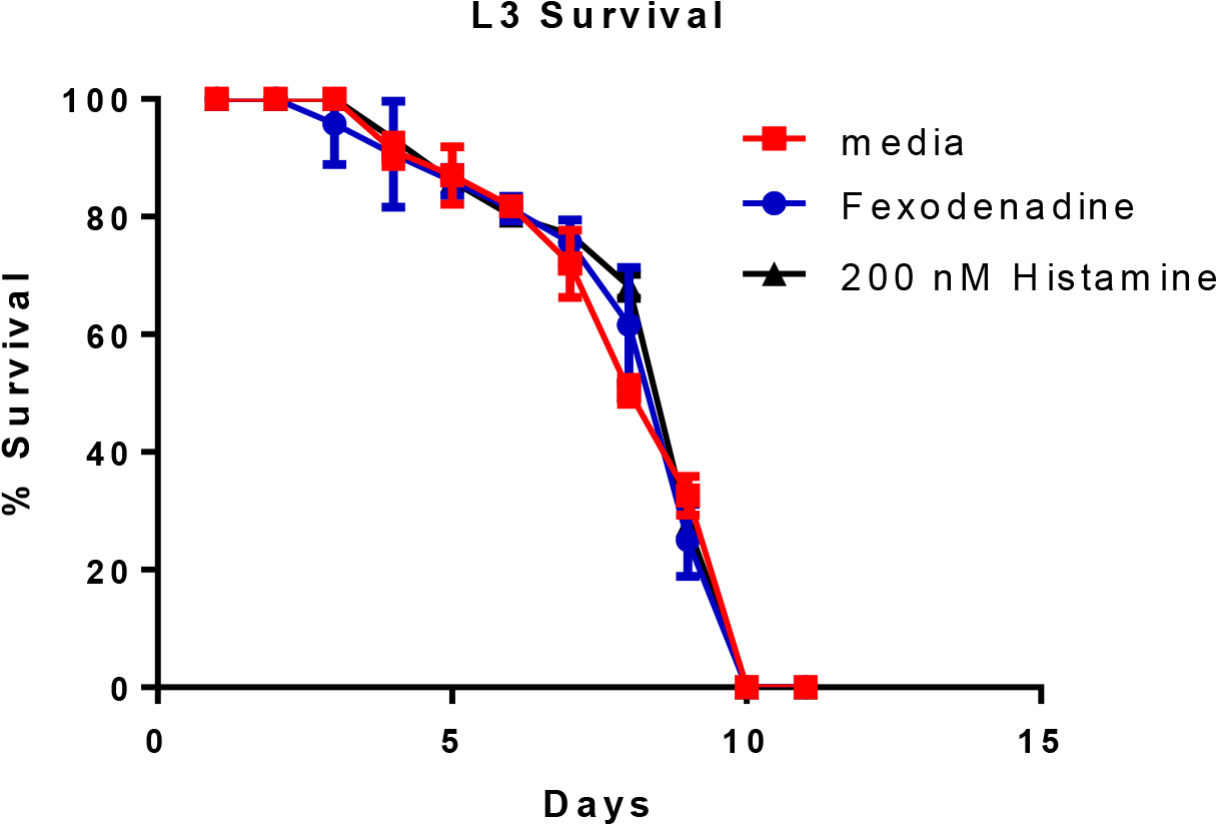
Fexofenadine is not directly toxic to *L*. *sigmodontis*. 200 L3s were cultured *in vitro* with daily addition of 200 nM histamine, 1mM fexofenadine, or media. Survival was assessed daily by visual inspection of motility. Data are combined from two independent experiments. Possible differences between groups at each timepoint were assessed by the Kruskal-Wallis test followed by Dunn Multiple comparisons.

Given the reduction in adult worm burden observed in HR1 treated mice, we next evaluated whether fexofenadine is directly toxic to *L*. *sigmodontis* worms and whether exogenous histamine enhances worm survival. To test this, L3 stage worms were cultured *in vitro* and supplemented daily with 200nM histamine, 10mM of fexofenadine, or media alone and assessed daily for survival. As seen in Fig 4, there were no observed differences in survival times between L3s supplemented with histamine, L3s supplemented with fexofenadine, and those supplemented with media (Fig 4). These data indicate that exogenous histamine does not directly enhance worm survival and that fexofenadine is not directly toxic to worm viability.

### Fexofenadine reduces antigen-specific IgE, IL-5, and IFN-γ responses

**Fig 5:**
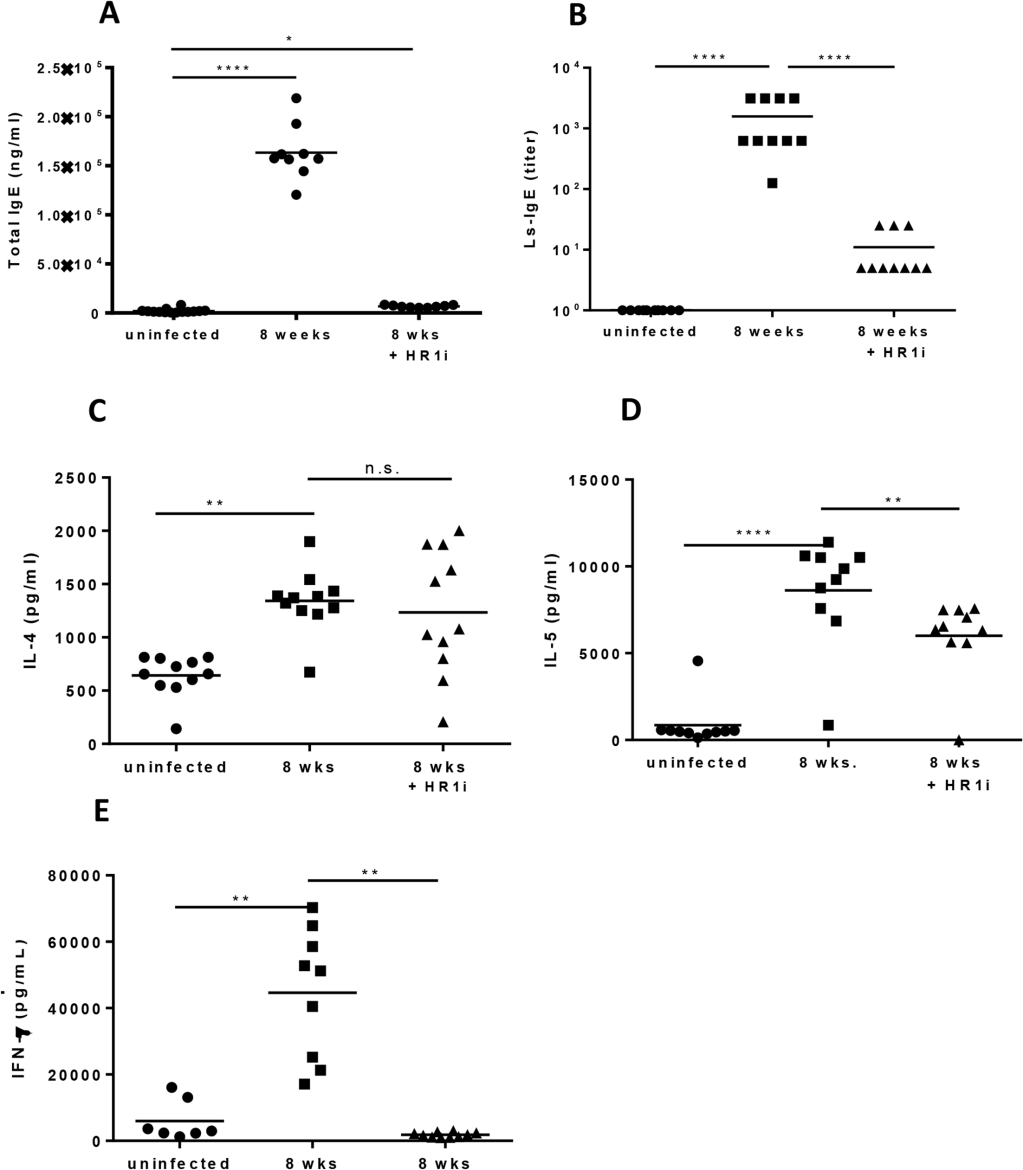
Immune modulation by histamine during *L*. *sigmondontis* infection. *(A)* Total IgE from mice treated with fexofenadine (HR1i) measured by ELISA. *(B)* Parasite specific (LsAg) IgE in mice treated with fexofenadine (HR1i) measured by ELISA. *(C*, *D*, *E)* IL-4, IL-5 and IFN-γ production by CD4^+^ cells from infected fexofenadine treated mice co-cultured with CD11c^+^ cells from naïve, age-matched mice and activated with anti-CD3/anti-CD28. Cells were cultured 1:10 (CD11c^+^:CD4^+^). Cytokine production was measured by ELISA. Data are from two combined experiments. *p<0.05, **p<0.01, ***p<0.001, ****p<0.0001, Kruskal-Wallis test followed by Dunn Multiple comparisons.

Because fexofenadine did not appear directly toxic to *L*. *sigmodontis* worms, we next evaluated whether H1R antagonism alters the immune response that develops during infection. To assess this, humoral and cellular immunological studies were conducted on infected mice treated with 8 weeks of fexofenadine. Both total and LsAg-specific IgE were significantly decreased in mice treated with fexofenadine compared to untreated controls (Fig 5A and 5B). In terms of cellular immune responses, parasite antigen-driven production of IL-5 and IFN-γ from splenocytes was also significantly reduced in fexofenadine treated mice (Fig 5D and 5E), whereas IL-4 production was not (Fig 5C). This suggests that signaling via HR1 may enhance both type 1 and type 2 immune responses.

### Fexofenadine-treated mice exhibit increased eosinophil numbers at site of adult worm infection

**Fig 6:**
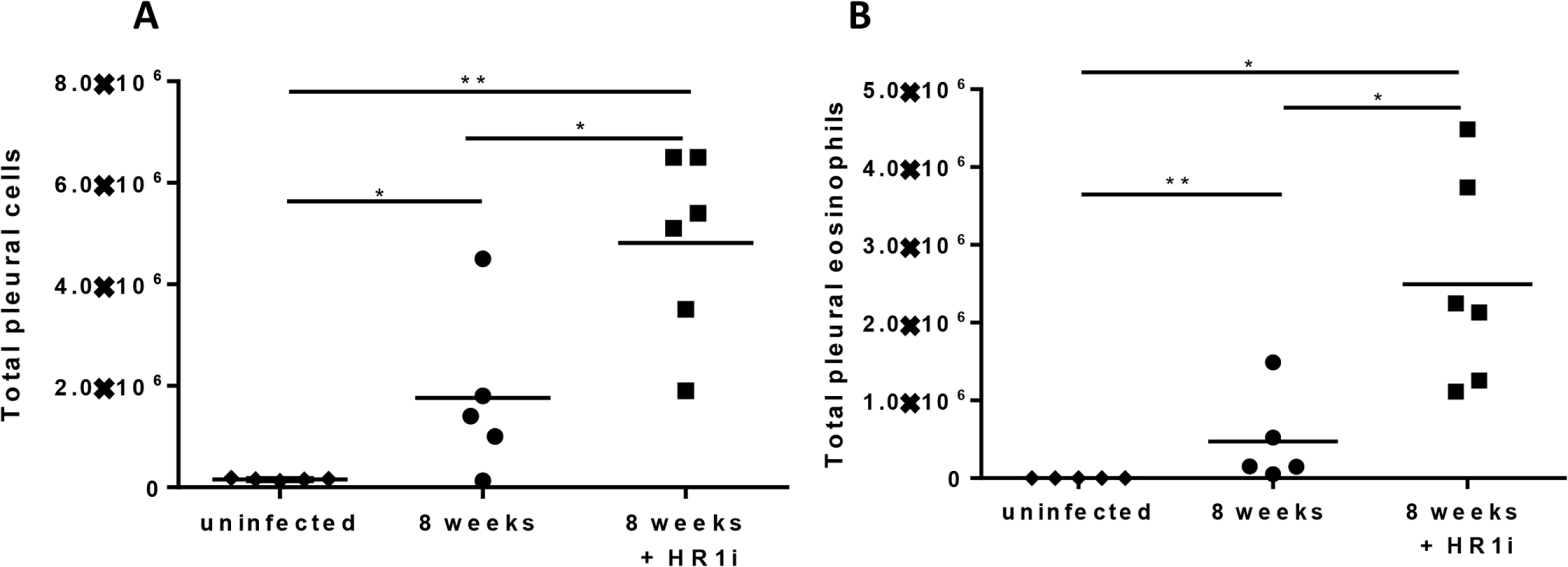
BALB/c mice treated with fexofenadine have an increase in pleural eosinophils. *(A)* Total pleural cells recovered from BALB/c and ΔGATA mice treated with fexofenadine (HR1i). *(B)* Total pleural eosinophils from BALB/c and ΔGATA mice treated with fexofenadine (HR1i) as determined by flow cytometry. *p<0.05, **p<0.01, Kruskal-Wallis test followed by Dunn Multiple comparisons.

In contrast to the decreases in IgE, IL-5, and IFN-γ, the cellular infiltrate present in the pleural cavity, the site where adult *L*. *sigmodontis* worms reside, was dramatically increased in fexofenadine treated mice. Whereas infection of untreated BALB/c mice resulted in a median of 1.7 x 10^6^ cells in the pleural space at study endpoint, fexofenadine-treated mice had 4.8 x 10^6^ cells (p = 0.0080, Fig 6A). Flow cytometric analysis revealed that eosinophils comprised over half of the cells in the pleural infiltrate of fexofenadine-treated mice, increasing in numbers from a median of 4.7 x 10^5^ cells in untreated infected mice to 2.5 x 10^6^ cells fexofenadine-treated infected animals (p = 0.0043, Fig 6B)

### Eosinophils are required for antihistamine mediated adult worm clearance

**Fig 7:**
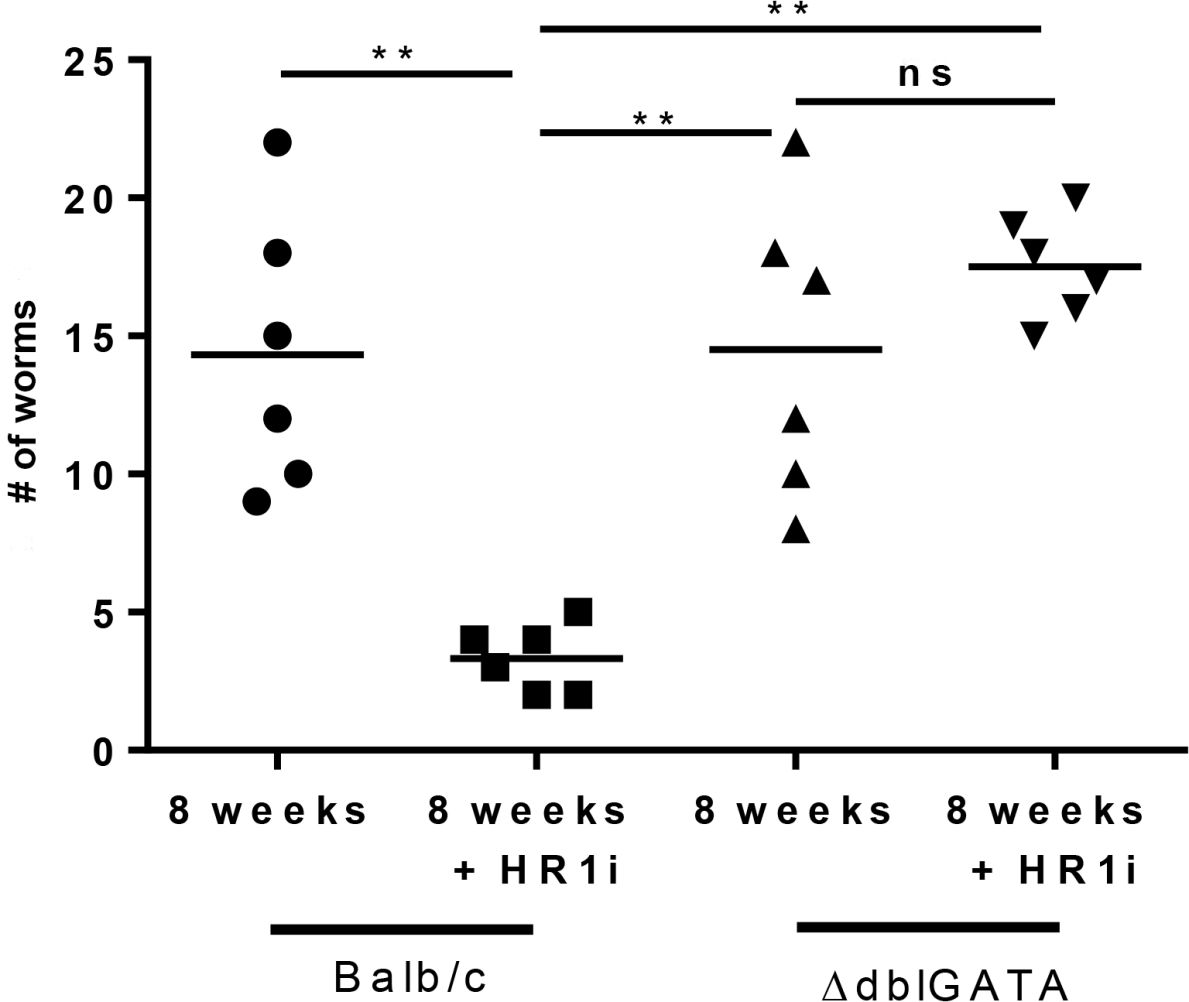
Fexofenadine-mediated worm clearance is dependent on eosinophils. Wild-type BALB/c and eosinophil deficient ΔdblGATA mice on a BALB/c background were infected with 40 L3-stage *L*. *sigmodontis* larvae and treated with fexofedine (HR1i) administered in drinking water for 8 wks. Control wild-type and eosinophil-deficient mice received no fexofenadine. Adult worm burden was determined in all groups at 8 wks. Data are combined from two independent experiments. ** p<0.01 by Kruskal-Wallis test followed by Dunn Multiple comparisons.

A number of studies utilizing the *L*. *sigmondontis* model have demonstrated a significant role for eosinophils in immune-mediated clearance of worms [14,15]. As fexofenadine increased eosinophil numbers at the site of adult worm infection, we next tested whether fexofenadine mediated worm clearance is dependent on eosinophils. Eosinophil deficient mice (ΔdblGATA) and background control BALB/c mice were infected with *L*. *sigmondontis*, treated for 8 weeks with HR1 antagonists, and euthanized at 8 weeks for enumeration of adult worm burden. In contrast to fexofenadine-treated wild type mice, eosinophil deficient mice administered fexofenadine had no reduction in adult worm burden (mean recovery 18) when compared to untreated ΔdblGATA controls (mean recovery 15) or BALB/c background controls (mean recovery 14) (Fig 7). To further assess the activity of eosinophils in antihistamine mediated worm clearance, BALB/c mice were infected, treated with HR1 antagonists for 8 weeks, and then euthanized. A pleural lavage was performed and ELISA used to detect eosinophil peroxidase (EPO) as evidence of eosinophil degranulation. Fexofenadine treated mice demonstrated significantly highler levels of EPO in the lavage fluid (S4 Fig). Taken together, these findings demonstrate that H1R blockade enhances worm clearance through an eosinophil-dependent mechanism.

## Discussion

In this study we found that histamine is released throughout filarial infection, that antihistamine therapy reduced IgE levels and increased eosinophilic responses at the site of infection, and that administration of fexofenadine, a HR1 blocker, enhances clearance of adult worms in an eosinophil-dependent manner.

Our first experiment was a time course study of circulating histamine levels to determine the kinetics of histamine release during primary filarial infection. As the t_1/2_ of histamine in blood is approximately 60s [16,17], blood levels are representative of ongoing histamine release. We found that histamine was released throughout the course of primary *L*. *sigmondontis* infection. The 1^st^ peak in circulating histamine occurred 30 minutes post infection in naïve mice. This finding has two important implications. First, as basophils and mast cells are the only cells carrying pre-formed histamine [18], it suggests that one or both of these cell types are activated within minutes of filaria infection. Early activation of these cells may be important for the shaping of the immune response to tissue invasive helminths. Second, early histamine release represents a non-specific mechanism of mast cell or basophil activation, since specific IgE is not present until weeks after infection. This is consistent with a number of studies that have demonstrated direct activation of basophils by helminth antigens (reviewed in [10]).

Timecourse studies next revealed that histamine release in infected mice peaks again at 8 weeks of infection. We speculate that the 8 week peak may be due to basophil and mast cell activation in response to circulating microfilariae, which appear starting 7 weeks post-infection. As detectable LsAg-specific IgE develops by 6 wks post-infection, this activation is likely occurring through IgE. This 2^nd^ peak is then followed by a decrease in circulating histamine, even though histamine decarboxylase message in blood cells increases throughout infection. This data is consistent with previous findings that basophils become hyporesponsive over time, requiring more signal to achieve activation [19]. Therefore, even though histamine synthesis continues throughout the course of infection, basophils and mast-cells are releasing less histamine in the chronic stages of infection.

Perhaps the most striking finding of this study is the significant reduction of adult worm burden at 8 weeks in mice treated with a HR1 antagonist. To determine the timing of worm clearance, infected mice were treated for 10, 35, or 56 days with fexofenadine and assessed for adult worm burden at 8 weeks. We found that the longer mice were treated with fexofenadine the greater the reduction in adult worm burden at 8 weeks and that treatment with fexofenadine resulted in a significant increase in encased worms in all groups. Previous studies have suggested a role for histamine in early larval invasion into the lymphatics [20]. Taken together these data indicate that that HR1 blockade enhances worm clearance at numerous stages of development.

As in vitro studies revealed that fexofenadine is not directly toxic to *Litomosoides sigmodontis*, we next evaluated whether fexofenadine augments immune responses directed against the parasite. Although helminth-specific IgE, IL-5 and IFNγ responses were all decreased in fexofenadine treated mice, eosinophil numbers at the site of worm infection were significantly elevated. Experiments with eosinophil deficient ΔdblGATA mice demonstrated that eosinophils were required for fexofenadine-mediated helminth clearance. In contrast to fexofenadine treated wild type mice, which had 80% fewer adult worms than wild type controls, fexofenadine treated ΔdblGATA mice exhibited no decrease in worm numbers. These results are consistent with prior studies suggesting eosinophils are key effector cells against helminths [21–23]. Studies utilizing the *L*. *sigmondontis* model have shown that mice deficient in eosinophil peroxidase or major basic protein, key eosinophil granule proteins, have significantly higher filarial worm burdens than wild type controls [14].

Prior studies [24] have shown that mice deficient in IL-5 produce neutrophilic rather than predominantly eosinophilic granulomas around *L*. *sigmodontis* worms. In our study, IL-5 production was likely not the driving mechanism for larger eosinophilic granulomas in the setting of H1R blockade as splenocytes from fexofenadine-treated mice demonstrated less IL-5 production than splenocytes from untreated infected animals. Together, the results of these papers and this study suggest that IL-5 is required for eosinophilic granuloma formation, and that fexofenadine enhances this process.

There are multiple mechanisms by which HR1 blockade may have enhanced eosinophil responses in this study. One possibility is that HR1 blockade may have enhanced eosinophil survival. Data from one *in vitro* study suggests histamine signaling reverses IL-5 afforded eosinophil survival [25]. A second hypothesis to explain increased eosinophil numbers at the site of worm infection is enhancement of eosinophil chemotaxis by blockade of HR1 signaling. Histamine is a known chemoattractant molecule for eosinophils [26–28], and the recently discovered [29] histamine receptor 4 (HR4) has been demonstrated to play a significant role in eosinophil chemotaxis and activation [30–35]. As such, it is possible that blockade of histamine signaling through HR1 enhances the effects of histamine through HR4. Alternatively, HR1 blockade may indirectly enhance eosinophil chemotaxis by increasing production of eosinophil chemotaxins or augmenting eosinophil sensitivity to such agents. Of note, dblGATA mice are deficient in basophils as well as eosinophils. Thus, it is possible that worm clearance in fexofenadine-treated mice is due to the action of basophils rather than eosinophils. However, as we have previously found that depletion of basophils does not alter adult worm numbers ([36], [37]), and as eosinophils are known to have the ability to kill adult filarial worms [14,38], we believe it is most likely that worm clearance induced by fexofenadine is through enhancement of eosinophil numbers at the site of infection.

One of the most interesting findings of this study is the observation that fexofenadine treatment caused significant reductions in circulating IgE levels and splenocyte production of IL-5 and IFNγ as well as increased numbers of eosinophils at the site of infection. While we can only speculate on the mechanisms underlying these apparently contrasting findings, we expect that it may be related 1) to the concentrations of histamine locally (at the site of infection) vs systemically, and 2) to unknown effects of histamine on the function of various immune effector cells. Another possibility is that decreased IgE levels and IL-5 production may have been due to the decreased adult worm burdens observed in fexofenadine-treated mice. The concentrations of histamine at different body sites during infection and the effects histamine has type 2 responses from B cells, T cells, macrophages, and dendritic cells will be the focus of future investigations. Another possibility is that decreased IgE levels and IL-5 production may have been due to the decreased adult worm burdens observed in fexofenadine-treated mice.

These findings demonstrate that histamine, in addition to its immediate proinflammatory effects, also functions to shape the immune response to helminth infections. The exact mechanisms by which this occurs are not yet clear. Histamine is known to alter the immunological function of a variety of cell types, including epithelial cells, granulocytes, T-cells, B-cells, and dendritic cells [3,39–41]. Investigations combining HR1 deficient mice with airway hyperresponsiveness models are mixed [42–45]. Whereas one showed decreases in type 2 cytokines, no changes in IFNγ, decreased IgE levels, and increased blood eosinophil numbers [42], another showed increases in type 2 cytokines, decreased IFNγ, and decreased bronchoalveolar lavage eosinophil numbers [43]. We believe the differences in these studies demonstrate the complex role histamine plays in shaping immune responses. The exact effects of histamine are likely dependent not only on the cell types involved, but also on the cytokine environment in which histamine is acting and on the repertoire of histamine receptors displayed by individual cells.

The results of our study may have some important clinical ramifications. Currently there is a worldwide effort to control and potentially eradicate lymphatic filariasis and onchocerciasis by repeated mass drug administration (MDA) of anti-filarial medications, especially diethylcarbamazine (DEC) [44]. A major factor limiting success of MDA is the inability of anti-filarial drugs to kill adult worms when given as a short course [45]. Since antifilarial medications primarily clear microfilariae, ongoing mass drug administration programs require repeated administration of antifilarial agents s for years until natural death of adult worms occurs. [46,47]. One of the interesting aspects of DEC therapy is that DEC does not appear sufficient on its own to kill filarial worms. Numerous studies have shown that DEC-mediated clearance of filariae is dependent in large part on the host immune response [48,49].Since we have shown that fexofenadine can augment immune clearance of adult filarial worms, we hypothesize that addition of fexofenadine to DEC or other antifilarial medications may result in better adult worm eradication than current regimens. Discovering a short course therapy that can successfully eliminate adult filarial worms would greatly increase our ability to control and eradicate filarial infections. Further elucidating the mechanisms by which fexofenadine decreases adult worm burdens, and investigating whether combining fexofenadine with current antifilarial medications enhances adult worm clearance, will be the focus of future studies.

## Supporting Information

S1 Fig Histamine levels are not elevated by peritoneal injection of RMPI or peritoneal lavage fluid. Age-matched BALB/c mice assessed for circulating histamine in plasma as measured by histamine ELISA. N.s = not significant by Mann-Whitney test.
(TIF)

S2 Fig Microfilariae load is not altered by HR1 blockade. Wild-type BALB/c were infected with 40 L3-stage *L*. *sigmodontis* larvae and treated with fexofenadine (HR1i) administered in water for 8 weeks. Control BALB/c mice received no fexofenadine. Microfilariae per mL of blood was determined at 8 weeks. n.s = not signficiant by Mann-Whitney test.
(TIF)

S3 Fig Male-to-female ratio is not altered by HR1 blockade. Wild type BALB/c were infected with 40 L3-stage *L*. *sigmodontis* larvae and treated with fexofenadine (HR1i) administered in water for 8 weeks. Control BALB/c mice received no fexofenadine. Male-to-female ratio was determined at 8 weeks. n.s. = not significant by Mann-Whitney test.
(TIF)

S4 Fig Balb/c mice treated with fexofenadine have an increase in pleural eosinophil peroxidase (EPO). Wild type BALB/c were infected with 40 L3-stage *L*. *sigmodontis* larvae and treated with fexofenadine (HR1i) administered in water for 8 weeks. Control BALB/c mice received no fexofenadine. EPO in pleural cavity was determined by pleural lavage followed by ELISA. ** p<0.01 by Kruskal-Wallis test followed by Dunn Multiple comparisons.
(TIF)
