# Correction: Histamine 1 Receptor Blockade Enhances Eosinophil-Mediated Clearance of Adult Filarial Worms

**DOI:** 10.1371/journal.pntd.0004034

**Published:** 2015-08-19

**Authors:** Ellen Mueller Fox, Christopher P. Morris, Marc P. Hübner, Edward Mitre

There are errors in the author affiliations. The affiliations should appear as shown here:

Ellen Mueller Fox^1^, Christopher P. Morris^1^, Marc P. Hübner^1,2^, and Edward Mitre^1^

^1^Department of Microbiology and Immunology, Uniformed Services University, Bethesda, MD 20815; ^2^Institute for Medical Microbiology, Immunology, and Parasitology, University Hospital Bonn, 53105 Bonn, German

## References

[1] Fox EM, Morris CP, Hübner MP, Mitre E (2015) Histamine 1 Receptor Blockade Enhances Eosinophil-Mediated Clearance of Adult Filarial Worms. PLoS Negl Trop Dis 9(7): e0003932 doi: 10.1371/journal.pntd.0003932 26204515 10.1371/journal.pntd.0003932 PMC4512699

