# Supplementary figures and images for: Histamine 1 Receptor Blockade Enhances Eosinophil-Mediated Clearance of Adult Filarial Worms
Source: PLoS Negl Trop Dis. 2015 Jul 23;9(7):e0003932. doi: 10.1371/journal.pntd.0003932 (PMC4512699; doi:10.1371/journal.pntd.0003932)

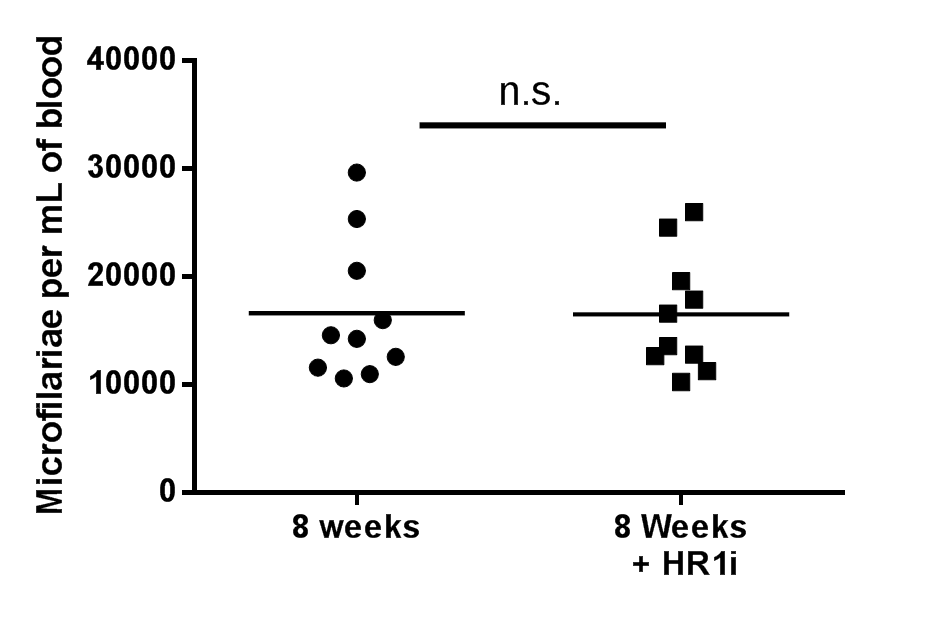

Supplement: S2 Fig — Wild-type BALB/c were infected with 40 L3-stage L. sigmodontis larvae and treated with fexofenadine (HR1i) administered in water for 8 weeks. Control BALB/c mice received no fexofenadine. Microfilariae per mL of blood was determined at 8 weeks. n.s = not signficiant by Mann-Whitney test. (TIF) [file pntd.0003932.s002.tif]

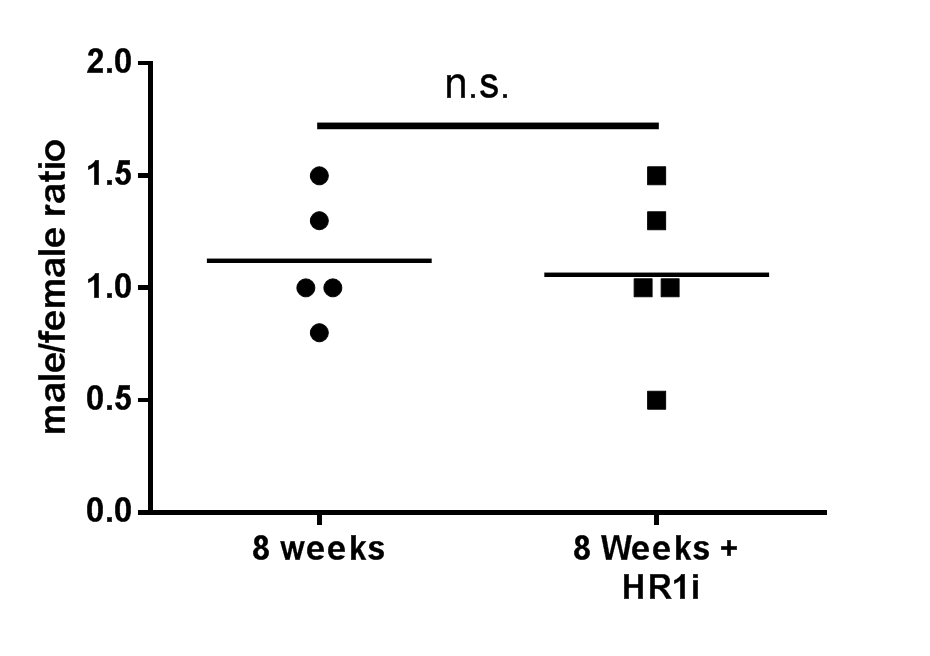

Supplement: S3 Fig — Wild type BALB/c were infected with 40 L3-stage L. sigmodontis larvae and treated with fexofenadine (HR1i) administered in water for 8 weeks. Control BALB/c mice received no fexofenadine. Male-to-female ratio was determined at 8 weeks. n.s. = not significant by Mann-Whitney test. (TIF) [file pntd.0003932.s003.tif]

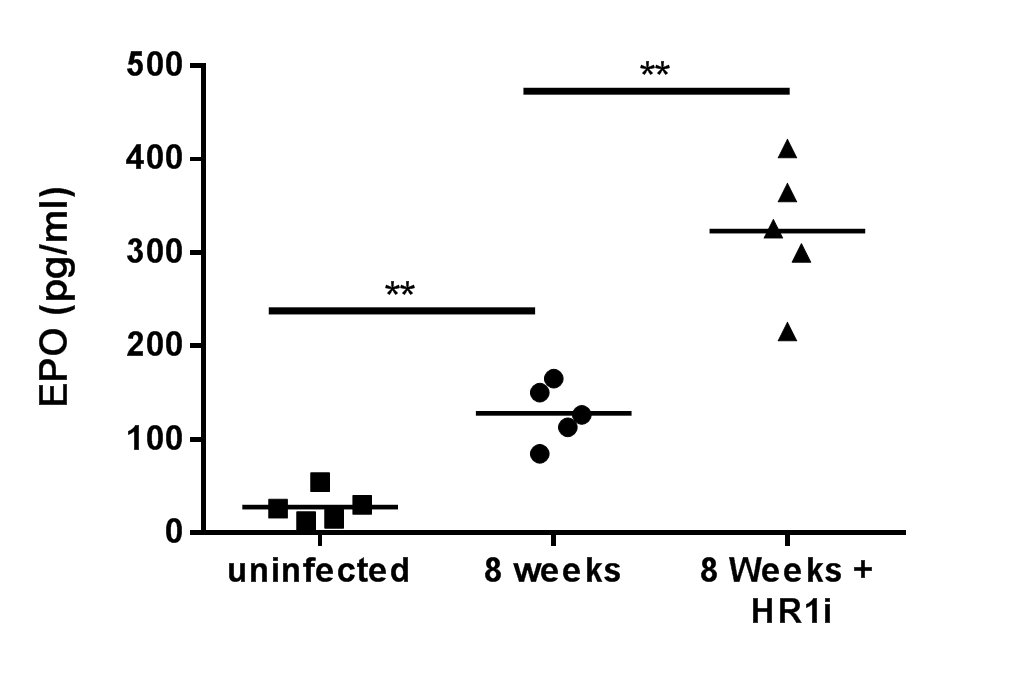

Supplement: S4 Fig — Wild type BALB/c were infected with 40 L3-stage L. sigmodontis larvae and treated with fexofenadine (HR1i) administered in water for 8 weeks. Control BALB/c mice received no fexofenadine. EPO in pleural cavity was determined by pleural lavage followed by ELISA. ** p<0.01 by Kruskal-Wallis test followed by Dunn Multiple comparisons. (TIF) [file pntd.0003932.s004.tif]
